# Biologic Treatment Modification Efficacy in Concurrent Inflammatory Bowel Disease and Ankylosing Spondylitis: A Retrospective Cohort Study at a Single Tertiary Center

**DOI:** 10.3390/jcm12227151

**Published:** 2023-11-17

**Authors:** Einat Savin, Niv Ben-Shabat, Asaf Levartovsky, Adi Lahat, Mahmud Omar, Omer Gendelman, Merav Lidar, Abdulla Watad, Shomron Ben-Horin, Uri Kopylov, Kassem Sharif

**Affiliations:** 1 Department of Medicine ‘B’, Zabludowicz Center for Autoimmune Diseases, Sheba Medical Center, Tel-Hashomer, Ramat Gan 52621, Israel; einat.shalom3@gmail.com (E.S.); nivben7@gmail.com (N.B.-S.); mahmudomar70@gmail.com (M.O.); omer.gendelman@sheba.health.gov.il (O.G.);; 2 Faculty of Medicine, Tel Aviv University, Tel Aviv 69978, Israelzokadi@gmail.com (A.L.); merav.lidar@sheba.health.gov.il (M.L.); shomron.benhorin@gmail.com (S.B.-H.); uri.kopylov@sheba.health.gov.il (U.K.); 3 Department of Gastroenterology, Sheba Medical Center, Tel-Hashomer, Ramat Gan 52621, Israel; 4 Rheumatology Unit, Sheba Medical Center, Tel-Hashomer, Ramat Gan 52621, Israel

**Keywords:** inflammatory bowel disease (IBD), ankylosing spondylitis (AS), management, biologics, clinical outcomes

## Abstract

Background: The link between ankylosing spondylitis (AS) and inflammatory bowel disease (IBD) is well-established, with concurrent prevalence estimates ranging from 5–10%. However, there are still significant gaps in our understanding, and a comprehensive treatment guideline for these co-diagnosed patients has yet to be established. Our objective was to explore patterns of treatment alterations following the diagnosis of AS in patients previously diagnosed with IBD, and vice versa. Additionally, we sought to determine how these modifications influence clinical outcomes in both conditions. Methods: This retrospective data-based cohort study included patients with coexisting IBD and AS that were diagnosed between the years 2009–2022 and were followed by the gastroenterology and the rheumatology units of the Sheba Medical Center, Israel. The data were extracted from the electronic health record and included demographic information, medication history, treatment modification at the time of second diagnosis, and the characteristics and activity of both IBD and AS at the index time and at the 3-month mark. Results: The study included a total of 68 patients, with a male predominance (40 patients, 59%). The median age was 43 years (IQR 31–55) and 78% had Crohn’s disease (CD). The median duration between the diagnosis of the first disease to the second one was 4 years (IQR 1–9.5). A significant proportion of patients (85%) underwent treatment modification at their second diagnosis. Out of the total cohort, 28% initiated biological therapy, 17.6% switched their biologic regimen, and 16.2% discontinued NSAIDS. Patients who underwent biologic modifications at time of the second diagnosis (the initiation/switch/augmentation of a concurrent regimen) experienced significantly higher rates of clinical improvement in either IBD or AS at the 90-day follow-up compared to patients who did not (68% vs. 32%, *p* = 0.004), and biologic modification was found to be an independent predictor for clinical improvement (OR 3.69, CI 1.08–12.58, *p* = 0.037). Conclusions: Our findings suggest that biologic therapy modification at the time of the second diagnosis was associated with a higher rate of improvement in AS/IBD at the 90-day follow-up.

## 1. Introduction

Inflammatory bowel disease (IBD), which encompasses Crohn’s disease (CD) and ulcerative colitis (UC), is a chronic inflammatory disease that impacts the gastrointestinal tract. Concomitant with IBD are various extraintestinal manifestations, with spondylarthritis (SpA) being the most predominant, manifesting as the axial or peripheral variety, or a combination of both involvements. [1]. Ankylosing spondylitis (AS) represents an advancing form of SpA, predominantly affecting the axial skeleton. It generally manifests before the age of 40 and exhibits a higher prevalence among males [2]. The diagnostic criteria for AS hinge on the clinical presentation of inflammatory back pain, often paired with radiographic indications of sacroiliitis, although the latter is not always obligatory. Common extra-articular manifestations include psoriasis, uveitis, and IBD [3,4]. The correlation between AS and IBD has been firmly established, with previous reports suggesting a shared genetic predisposition [1,5,6]. The existence of overlapping treatments further supports this correlation [7]. The estimated prevalence of coexisting AS and IBD ranges between 5–10% [8], and radiologic evidence of sacroiliitis is observed in 20–50% of patients with IBD [9]. Additionally, in patients with AS, endoscopic macroscopic bowel inflammation is reported in 14–37% of patients, whereas microscopic inflammation is noted in approximately 50–58% of cases [8,10,11,12].

The concurrent management of AS and IBD presents challenges, with many aspects of their combined treatment still remaining ambiguous or unclear. Even though there are overlapping pathogenic mechanisms between these conditions, distinct differences in the therapeutic approaches are evident. Non-steroidal anti-inflammatory drugs (NSAIDs), often the first-line therapy for AS, should be used judiciously in IBD patients. On the other hand, systemic corticosteroids, commonly used during IBD flare-ups, have shown limited effectiveness in treating AS. [13,14]. Secukinumab, a monoclonal antibody that targets interleukin-17 (IL-17), has shown significant efficacy in treating AS [15]. Nonetheless, its use has been linked to the onset of new IBD cases. [16]. Conversely, ustekinumab, an inhibitor of IL-12/23, has demonstrated effectiveness in IBD [17], but has been proven ineffective for AS [18]. Anti-tumor necrosis factor (TNF) agents are frequently the preferred therapeutic choice for both AS and IBD [19]. However, etanercept, among these agents, has been shown to exacerbate IBD symptoms [20,21]. Broadly speaking, janus kinase (JAK) inhibitors (JAKi) represent an additional therapeutic option for patients with both IBD and AS [13,22]. 

Given the current gaps in the literature, investigating the temporal dynamics of the association between AS and IBD, analyzing the treatment strategies throughout the follow-up period, and assessing the outcomes of both conditions offer valuable insight for optimizing patient management with concurrent disease entities. Hence, we aimed to investigate a treatment modification pattern subsequent to the recent diagnosis of AS in patients with IBD, or a novel IBD diagnosis in patients with AS, along with the impact on the clinical outcomes of both conditions. 

## 2. Methods

### 2.1. Study Design and Population

In this retrospective cohort study, data were extracted from the medical records within the Department of Gastroenterology and Rheumatology unit at the Sheba Medical Center, the largest tertiary center in Israel. The patient electronic medical records encompassed data from hospital admissions, specialized clinical encounters, endoscopic procedures, and imaging tests performed as part of the patient’s care within the hospital. For this study, the criteria for eligibility were as follows: patients aged 18 years and older who had, in their medical record, a diagnosis of IBD and AS, with the later diagnosis occurring between the years 2009–2022. The identification of IBD and AS cases was based on the International Classification of Diseases (ICD-9) coding, specifically CD (555.0–555.2, 555.9), UC (556), and AS (720.0). Patients not receiving specialized care from both the Gastroenterology and Rheumatology departments at the Sheba Medical Center were excluded from the study. Additionally, individuals without confirmed disease documentation in their medical records, or those with a follow-up period of less than 3 months after their secondary diagnosis, were also excluded. The starting point for follow-up, or the index date, was determined as the date when the secondary condition (either IBD or AS) was diagnosed. The primary exposure was considered an initiation of biological therapy, an augmentation of a concurrent biological therapy, or switching from one biological therapy to another, all of which occurring during index date. The primary outcome was clinical improvement at 3 months as specified below.

### 2.2. Outcomes and Measures 

Disease activity was determined using the Simple Clinical Colitis Activity Index score (SCCAI) for UC and the Harvey–Bradshaw index (HBI) for CD. For AS disease activity, the Bath Ankylosing Spondylitis Disease Activity Index (BASDAI) was used. Disease activity levels for BASDAI were categorized as low (BASDAI < 2), moderate (2 ≤ BASDAI < 4), and high (BASDAI ≥ 4), utilizing previously described cut-off values [23]. Clinical severity for CD was defined as remission (HBI ≤ 4), mild (5 ≤ HBI ≥ 7), moderate (8 ≤ HBI ≥ 16), and severe (HBI ≥ 17), adhering to acceptable cut-off values [24,25]. Similarly, UC severity was defined using the following acceptable cut-off values of SCCAI: remission (SCCAI ≤ 2), mild (SCCAI = 3 to 5), moderate (SCCAI between 6 and 11), and severe (SCCAI ≥ 12) [26]. Clinical response was defined as a decline > 3 points in HBI, SCCAI, or BASDAI. Similarly, disease improvement was characterized by a reduction in the severity of the disease for each specific condition. A decrease in disease severity—such as a shift from moderate to mild, from mild to remission, or similar transitions—was considered an indication of improvement. Comparison of IBD and AS improvement rates three months after biologic modification, stratified by first diagnosis, was explored, as well as the improvement of either IBD or AS improvement at 3 months at follow-up visit.

### 2.3. Other Variables

The following data were extracted from the electronic health record of Sheba Medical Center:

1. Demographic information: This includes age, gender, year of diagnosis for both IBD and AS, and smoking status. 

2. Extraintestinal manifestation: This includes psoriasis, uveitis, erythema nodosum, and peripheral arthritis. 

3. IBD Characteristics: This includes CD/UC phenotype, extension, and history of IBD surgeries. 

4. Medication History:

- IBD-related medications: This includes 5-aminosalicylic acid (5-ASA), corticosteroids, and immunomodulators like azathioprine, mercaptopurine, and methotrexate.
- AS-related medications: This includes NSAIDs, sulfasalazine, and methotrexate.
- Biologicals agents for either AS or IBD treatment: This includes anti-TNF agents, tofacitinib, ustekinumab, secukinumab, and certolizumab pegol.

5. Laboratory Values: This includes C-reactive protein and calprotectin, at the initial index time and at a 3-month follow-up.

6. Treatment modification: Information regarding treatment adjustments made at the initial index time was extracted from patient records during their visits to both the gastroenterologist and rheumatologist. This includes 5-ASA initiation or augmentation, immunomodulator initiation, NSAID discontinuation, and biological modification (initiation, augmentation, or switching). 

### 2.4. Statistical Analysis

Categorical variables were reported as n (%), while continuous variables were reported as the median and interquartile range (IQR). The Chi-square test and Fisher’s exact test were employed as suitable methods for drawing comparisons between the groups. All tests were two-tailed, with *p*-values < 0.05 considered to be statistically significant. Binary logistic regression model was used to identify predictors for improvements. First, we used a univariate model examining each selected variable individually, followed by a multivariate model including variables found to be significant in the univariate model. Results were reported as odds ratio (OR) and the 95% confidence interval (CI). 

Data analysis was performed using IBM SPSS Statistics version 26 (IBM, Armonk, NY, USA).

## 3. Results

**Figure 1:**
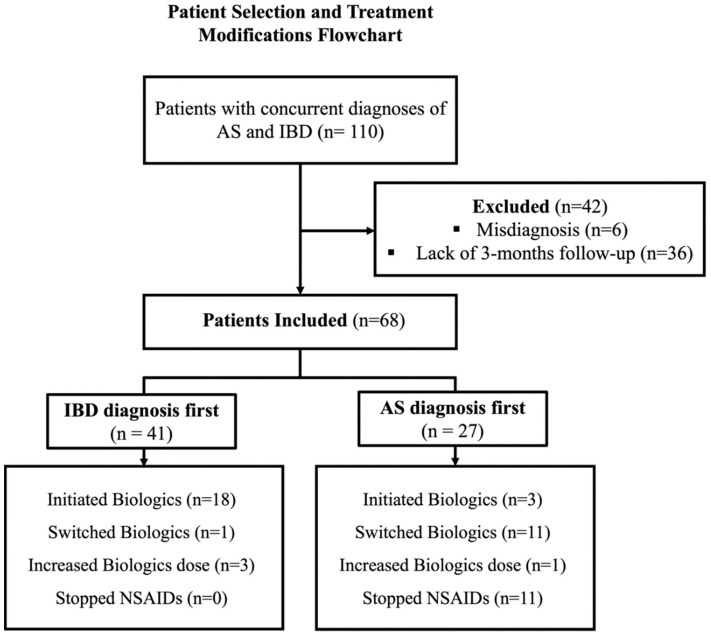
Patient selection flow chart.

Of the 110 patients with documented co-diagnoses of AS and IBD in their clinical records, 42 were excluded from the study. Of these, 6 patients were inappropriately assumed in their medical record to have IBD or AS, while 36 did not have a minimum follow-up duration of 3 months with either a gastroenterologist or rheumatologist after their secondary diagnosis. The final study cohort included a total of 68 patients with a male predominance (40 patients, 59%), with a median age of 43 (IQR 31–55), and 78% (53 patients) had CD. Baseline characteristics and treatment modification at the time of the second diagnosis are reported in Table 1, Figure 1.

**Figure 2:**
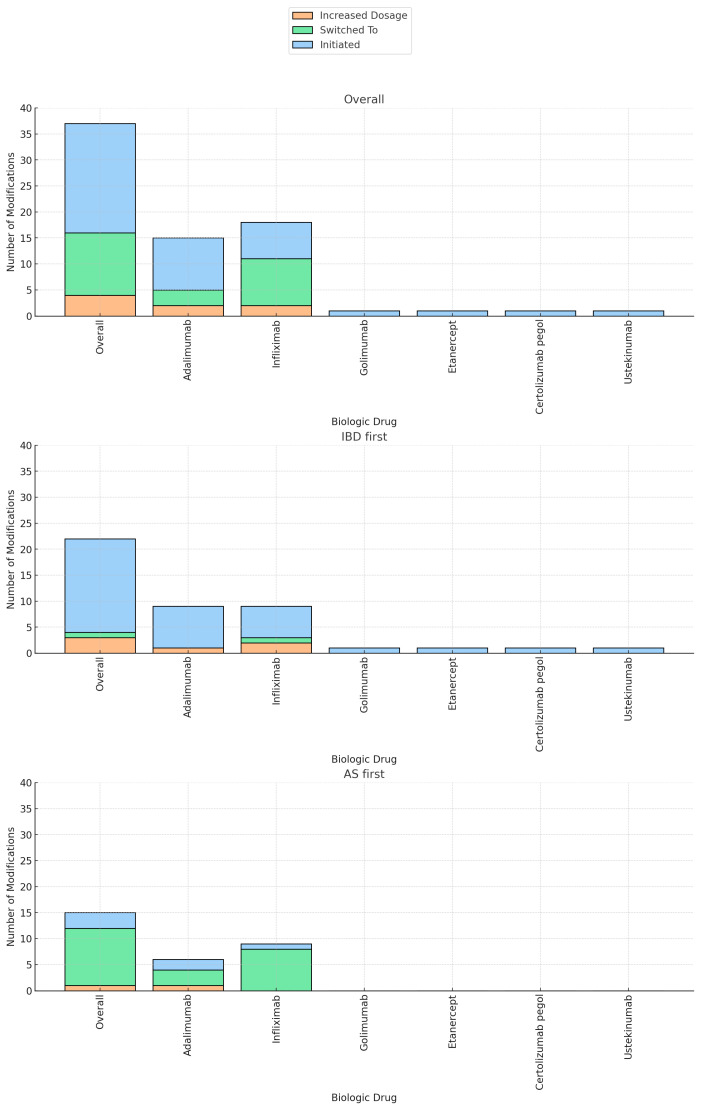
Biologic treatment modifications by group and specific drug.

The majority of patients (58 patients, 85%) underwent treatment modification at the time of the second diagnosis. Out of the total cohort, biologic therapy was initiated in 28% of the patients (21 patients), immunomodulators in 9% (6 patients), and 5-ASA in 6% (4 patients). NSAIDS was stopped in 16.2% of the patients (11 patients). The current biologic regimen was augmented in 6% of the patients (4 patients) and the biologic regimen was switched in 17.6% (12 patients) (Table 1). Biologic therapy was swapped out-of-class in 9/12 patients (etanercept/secukinumab to anti-TNF) and was changed intraclass in 3/12 patients (anti-TNF agents). Biologic therapy was predominantly initiated in patients who received an initial diagnosis of IBD, compared to those with a primary diagnosis of AS (18/41 patients vs. 3/27 patients, *p* < 0.01). Specifically, the initiated agents were adalimumab (10/21 patients), infliximab (7/21 patients), golimumab (1/21 patients), etanercept (1/21 patients), certolizumab pegol (1/21 patients), and ustekinumab (1/21 patients). Conversely, switching to a new biologic agent and the discontinuation of NSAIDs were more commonly observed in patients initially diagnosed with AS compared to the IBD group (41% vs. 2%, *p* < 0.01, 41% vs. 0%, *p* < 0.01, respectively) (Table 2, Figure 2).

In comparing patients subjected to biologic modifications (be it the initiation, switching, or augmentation of the concurrent regimen), it was observed that the proportion of males was significantly greater in the group that underwent such biologic alterations. (73% vs. 42%, *p* = 0.01). In addition, the prevalence of left-sided/extensive UC was higher in the patients who underwent biologic modification compared to patients who did not (100% vs. 67%, *p* = 0.05) (Table 3).

**Figure 3:**
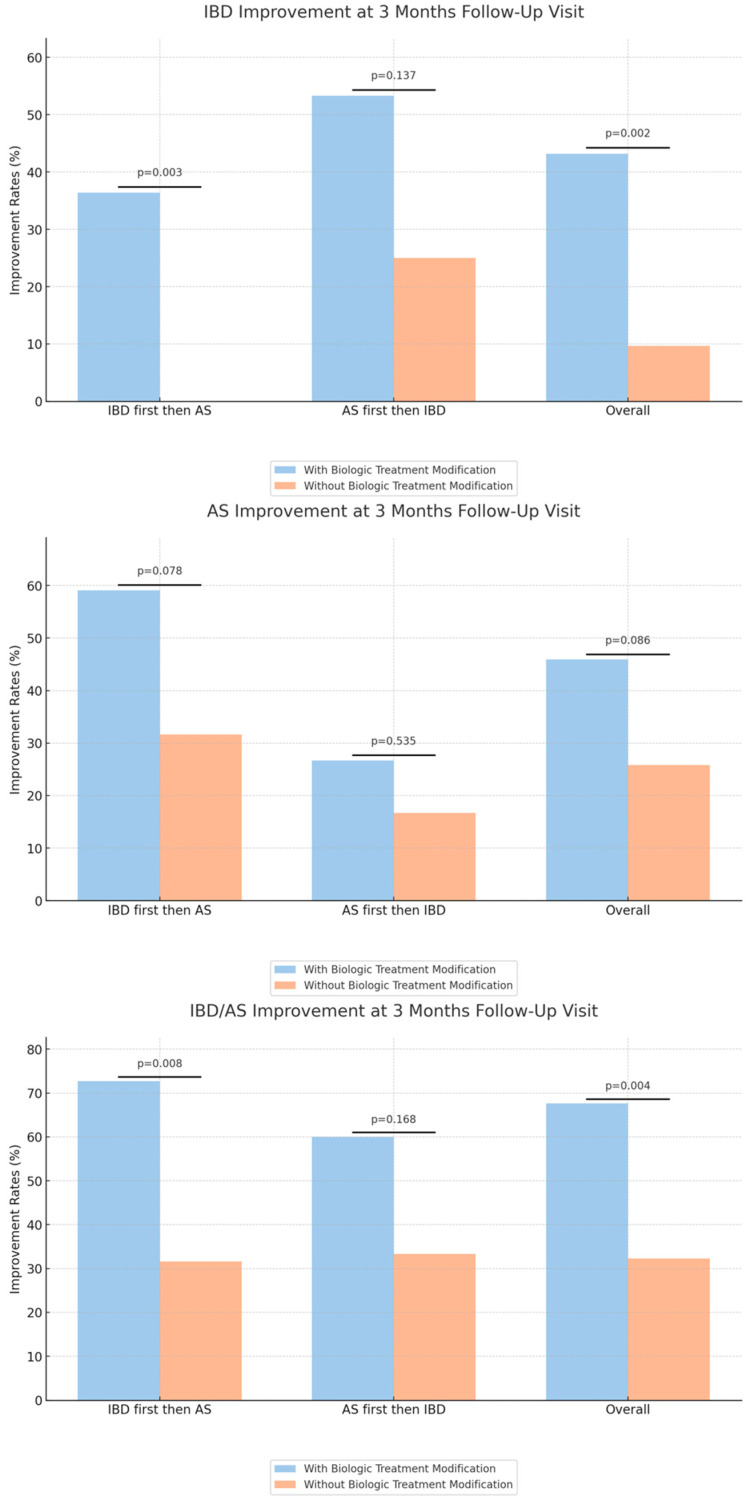
Comparison of improvement rates across cohorts.

Interestingly, patients who underwent biologic modifications experienced significantly higher rates of clinical improvement in either IBD or AS at the 3-month follow-up compared to patients who did not (68% vs. 32%, *p* = 0.004). However, the improvement in IBD was more pronounced than in AS, with rates of 43% IBD improvement among patients with biologic modification compared to 10% among the other group (*p* = 0.002) (Table 4, Figure 3).

We also evaluated the improvement outcomes for both IBD and AS at the 3-month mark, categorizing by the biologic modification and initial diagnosis subgroups. Within the group of patients with an initial diagnosis of IBD, patients who underwent biologic modification had a higher rate of IBD and either IBD or AS improvement, compared to patients who did not have biologic modification (36% vs. 0%, *p* = 0.003, 73% vs. 32%, *p* = 0.008, respectively). 

**Figure 4:**
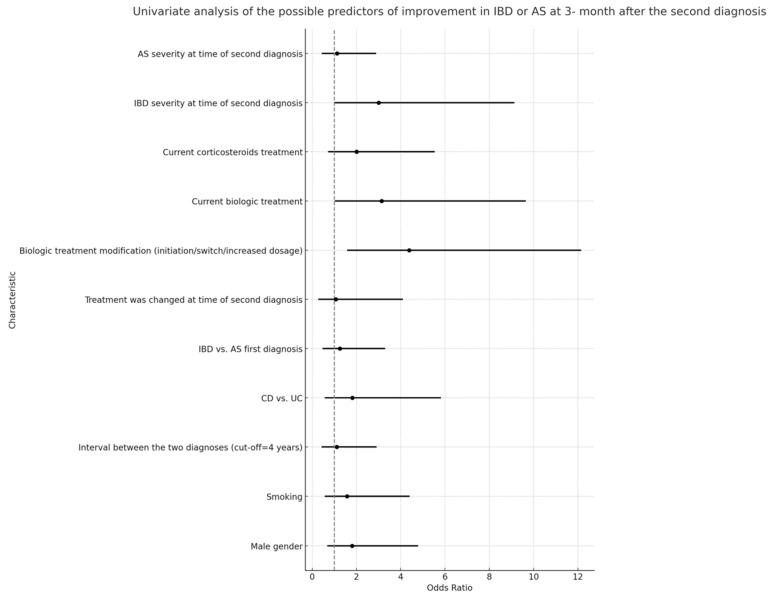
Univariate analysis of predictors for IBD or AS improvement three months after second diagnosis, including all listed characteristics.

**Figure 5:**
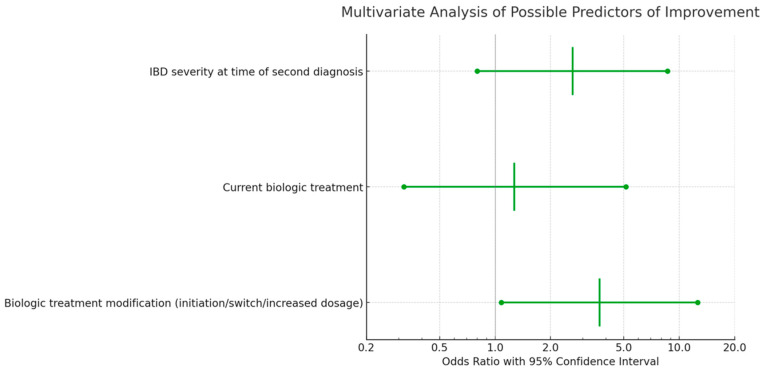
Multivariate analysis of predictors for IBD or AS improvement three months after second diagnosis, including all listed characteristics.

Multivariable logistic regression analyses indicated that the severity of IBD at the time of the secondary diagnosis was not correlated with a significant improvement in either IBD or AS at the 3-month follow-up (Figure 4 and Figure 5). However, a notable association was observed between a higher rate of improvement in AS/IBD and biologic treatment modifications made at the time of the second diagnosis (OR 3.69, CI 1.08–12.58, *p* = 0.037), as detailed in Table 5. 

## 4. Discussion

Our retrospective study uniquely examines the clinical trajectory of patients initially diagnosed with IBD or spondyloarthropathy who later received a second diagnosis, with a specific emphasis on the effects of biologic therapy adjustments. The observed relationship between AS and IBD, both as inflammatory diseases, provides valuable insights into potential shared pathophysiological mechanisms, with our results showing a concurrent improvement of both conditions upon the initiation or modification of biologic therapy, which underscores the need for integrated and comprehensive management strategies for these patients.

Looking at the initial cohort results, the data reveal distinct therapeutic patterns contingent on whether the initial diagnosis was IBD or AS. Treatment modifications at the time of the second diagnosis were markedly more prevalent among those first diagnosed with AS (96.3%) compared to those initially diagnosed with IBD (78%). This indicates a more aggressive or prompt adjustment in the therapeutic regimen for patients who originally presented with AS.

The use of NSAIDs showed a striking difference, as 40.7% of the AS-first cohort had NSAIDs as part of their treatment regimen. This cessation might reflect the known risks of NSAIDs in exacerbating IBD symptoms, or the initiation of more targeted therapies.

Conventional NSAIDs have been associated with triggering the onset of IBD, exacerbating existing conditions, and causing gastrointestinal complications. Various studies support a link between NSAID usage and increased hospital admissions for IBD flares [27,28,29,30,31]. On the other hand, selective COX2 inhibitors, designed to cause less gastrointestinal toxicity, have shown mixed results in their impact on IBD [32,33]. While some studies suggest that these inhibitors are safe and even beneficial for IBD patients, others report exacerbations and adverse effects such as abdominal pain and bloody stool.

Interestingly, biologic initiation was more frequently observed in the IBD-first cohort (43.9%) as opposed to the AS-first group (11.1%). Conversely, biologic switching was substantially more common among AS-first patients (40.7%) than those first diagnosed with IBD (2.4%). This might be explained by the fact that a higher proportion of AS patients were on biologic therapy versus IBD patients. These variations suggest that patients initially diagnosed with IBD might be more inclined to commence biologics, while those with an initial AS diagnosis might be more likely to switch between them. In essence, the sequence of disease diagnosis appears to significantly influence subsequent therapeutic adjustments, particularly in the utilization of biologics and NSAIDs [27].

Another notable observation from our results is the marked difference in improvement rates among patients who had their biologic treatment modified following the second diagnosis. Overall, a significant 67.6% of these patients demonstrated improvement compared to 32.3% who did not undergo a treatment modification (*p* = 0.004). When the sequence of diagnosis was considered, for those diagnosed with IBD followed by AS, a striking 72.7% showed improvement when the treatment was modified, highlighting the potential benefits of therapy alteration.

However, the sequence of diagnosis seems to play a role. While treatment modification after an IBD diagnosis seemed particularly efficacious, the same cannot be readily concluded for those diagnosed with AS followed by IBD. Such differences can be attributed to a myriad of reasons, including disease biology, the differential impact of biologics on each condition, or the stage at which the disease was diagnosed and, subsequently, treated.

The significance of biologic therapy emerges from the univariate analysis where patients on a current biologic treatment demonstrated an odds ratio of 3.142 for improvement, which was statistically significant (*p* = 0.046). Interestingly, the severity of IBD at the time of the second diagnosis almost reached statistical significance (*p* = 0.053) in relation to improvement, which might suggest that the timing of the therapy modification in relation to disease severity is crucial.

Several baseline characteristics, such as the male gender, smoking status, and the interval between diagnoses, did not significantly influence the outcomes. This is in contrast to some previous studies that have indicated, for instance, that the male gender might have a bearing on the IBD course [34]. It is imperative to consider our study’s unique cohort and design, which might contribute to these observed discrepancies. 

IBD and spondyloarthropathies, although primarily affecting distinct systems of the body, exhibit significant clinical and genetic overlap [19]. Gut inflammation indicative of IBD is observed in approximately 50–60% of AS patients, often without overt clinical symptoms. Both disorders have been linked to abnormalities in the IL-23 and Th17 cell axis, which are pivotal in regulating gut immune responses [35]. Anomalies in these pathways can instigate autoimmune reactions in the intestines and joints. The gut–joint axis theory further adds to this discourse, suggesting that microbial dysbiosis in the gut, a known factor in IBD pathogenesis, might also contribute to the onset of spondyloarthropathy. This theory postulates that certain gut bacteria can incite inflammatory reactions in genetically susceptible individuals, leading to inflammation in remote anatomical regions, such as the spine [36].

The genetic aspect of the association between these conditions is underlined by the HLA-B27 gene. While this gene is present in about 5–10% of the Caucasian population, its prevalence rises dramatically among AS patients, with around 90% exhibiting this gene. Interestingly, this gene is also overrepresented in IBD patients, especially those presenting with joint symptoms, hinting at a shared genetic predisposition [36].

The findings from our retrospective study underscore the potential interplay between biologic therapy modification and clinical outcomes in concurrent IBD and AS treatment. Notably, individuals diagnosed with IBD followed by AS, who underwent alterations in their biologic treatment regimen, experienced a marked improvement in their clinical response and remission rates compared to those without any therapeutic modifications. The overarching implications of the study suggest that biologic therapy adjustments might be a pivotal element in optimizing clinical outcomes for patients diagnosed with both conditions. It underscores the importance of individualized treatment strategies tailored to each patient’s unique needs, particularly when managing two intricately connected conditions like IBD and AS.

These insights offer a compelling reason for a synergized approach to patient care. By fostering collaboration between gastroenterologists and rheumatologists, there lies a promise to refine therapeutic strategies further. Such integrated approaches could potentially elevate the overall standard of care, ensuring that patients grappling with the dual challenges of IBD and AS receive the most effective treatments tailored to their unique clinical trajectories.

Our study has several strengths and limitations. Our main strength is the lack of previous studies investigating the area of interest. Several limitations of our strategy must be appreciated. Firstly, our study, being retrospective, could be affected by biases inherent to such designs. Second, our cohort, while spanning a decade, was based on a single center’s data, limiting generalizability. Moreover, while we excluded patients with less than three months of follow-up, a longer observational period might offer deeper insights into the long-term implications of biologic therapy modifications.

In conclusion, our findings shed light on the pivotal role biologic therapy modification can play in concurrently managing IBD and AS, especially when IBD precedes AS. The choice and timing of biologics, aligned with the nuanced understanding of both conditions, can lead to better patient outcomes. Further multicentric, prospective studies are warranted to validate our observations and offer clearer guidelines on the optimal therapeutic approach for this dual-diagnostic cohort.

## Figures and Tables

**Table 1:** Baseline cohort characteristics.

Cohort Characteristics	Entire Cohort (n = 68)
Male gender	40 (58.8)
Age, years	43.0 (31–55)
Smoking, current or former	22 (32.3)
Age at IBD diagnosis, years	27.0 (22.0–39.0)
Interval between the two diagnoses, years	4.0 (1.0–9.5)
CD	53 (77.9)
CD extent, n (% of CD patients)	
L1 (ileal)	33 (62.3)
L2 (colonic)	6 (11.3)
L3 (ileo-colonic)	14 (26.4)
Perianal disease	7 (13.2)
CD phenotype, n (% of CD patients)	
B1 (non-structuring non-penetrating)	42 (79.2)
B2 (structuring)	6 (11.3)
B3 (penetrating)	5 (9.4)
UC extent, n (% of UC patients)	
E1 (proctitis)	2 (2.9)
E2 (left-sided)	5 (7.4)
E3 (extensive)	8 (11.8)
Clinical extraintestinal manifestation	
Psoriasis	3 (4.4)
Uveitis	7 (10.3)
Peripheral arthritis	16 (23.5)
CRP at time of second diagnosis	18.0 (7.2–49)
IBD activity at time of second diagnosis	
Low	23 (33.8)
Moderate	25 (36.8)
High Very high	19 (27.9) 1 (1.4)
AS activity at time of second diagnosis	
Low	5 (7.4)
Moderate	27 (39.7)
High	36 (52.9)
Previous biologic therapy	17 (25.0)
Ongoing treatment during second diagnosis	
Biologic regimen	26 (38.2)
Corticosteroids	6 (8.8)
5-ASA	14 (20.6)
Immunomodulators (azathioprine/mercaptopurine/MTX)	8 (11.8)
NSAIDS	12 (17.6)
Treatment modification at time of second diagnosis	58 (85.3)
5-ASA initiated/increased dosage	4 (5.9)
Immunomodulators (azathioprine/mercaptopurine/MTX) initiated	6 (9.1)
NSAIDS stopped	11 (16.2)
Biologic initiated	21 (28.4)
Biologic switched	12 (17.6)
Biologic increased dosage	4 (5.9)

Data are n (%) or median (IQR). *p*-value < 0.05 are bold. IBD = Inflammatory bowel disease. CD = Crohn’s disease. UC = Ulcerative colitis. 5-ASA = 5-aminosalicylic acid. NSAID = Non-steroidal anti-inflammatory drugs. CRP = C-reactive protein. AS = Ankylosing spondylitis.

**Table 2:** Baseline characteristics stratified by the patient’s initial diagnosis.

Cohort Characteristics	IBD First Diagnosis (n = 41)	AS First Diagnosis (n = 27)	*p*-Value
Male gender	24 (58.5)	16 (59.3)	0.57
Age, years	43.0 (35–56)	36.0 (25–48)	**0.04**
Smoking, current or former	15 (36.5)	7 (25.9)	0.50
Age at IBD diagnosis, years	27.5 (19.2–39.0)	27.0 (22.0–39.0)	0.79
Interval between the two diagnoses, years	6.0 (2.0–11.5)	3.0 (1.0–6.0)	**0.03**
CD	28 (68.3)	25 (92.6)	**0.02**
CD extent, n (% of CD patients)			0.28
L1 (ileal)	16 (57.1)	17 (68.0)	
L2 (colonic)	5 (17.9)	1 (4.0)	
L3 (ileo-colonic)	7 (25)	7 (28.0)	
Perianal disease	6 (21.4)	1 (4.0)	0.10
CD phenotype, n (% of CD patients)			0.25
B1 (non-structuring non-penetrating)	20 (71.4)	22 (88.0)	
B2 (structuring)	5 (17.9)	1 (4.0)	
B3 (penetrating)	3 (10.7)	2 (8.0)	
UC extent, n (% of UC patients)			0.25
E1 (proctitis)	1 (2.4)	1 (3.7)	
E2 (left-sided)	5 (12.2)	0	
E3 (extensive)	7 (17.1)	1 (3.7)	
Clinical extraintestinal manifestation			
Psoriasis	2 (4.9)	1 (3.7)	0.81
Uveitis	4 (9.8)	3 (11.1)	0.85
Peripheral arthritis	5 (12.2)	11 (40.7)	0.78
CRP at time of second diagnosis	14.5 (7–37.5)	22.6 (11.3–66)	0.48
IBD activity at time of second diagnosis			0.09
Low	20 (48.8)	3 (11.1)	
Moderate	13 (31.7)	12 (44.4)	
High Very high	8 (19.5) 0	11 (40.7) 1(3.7)	
HBI at time of second diagnosis	5.0 (2.0–7.0)	2.0 (1.0–6.0)	0.23
SCCAI at time of second diagnosis	7.0 (5.0–8.0)	5.5 (4.3–6.8)	0.22
AS activity at time of second diagnosis			**<0.01**
Low	0 (0)	5 (18.5)	
Moderate	11 (26.8)	16 (59.3)	
High	30 (73.2)	6 (22.2)	
Previous biologic therapy	9 (22).0	8 (29.6)	0.57
Ongoing treatment during second diagnosis			
Biologic regimen	12 (29.3)	14 (51.9)	0.07
Current corticosteroids therapy	3 (7.3)	3 (11.1)	0.67
Current 5-ASA therapy	14 (34.1)	0 (0)	N/A
Current immunomodulatory therapy (azathioprine/mercaptopurine/MTX)	7 (17.0)	1 (3.7)	N/A
Current NSAIDS therapy	0 (0)	12 (44.4)	N/A
Treatment modification at time of second diagnosis	32 (78)	26 (96.3)	**0.04**
5-ASA initiated/increased dosage	3 (7.3)	1 (3.7)	0.48
Immunomodulators (azathioprine/mercaptopurine/MTX) initiated	3 (7.7)	3 (11.1)	0.64
NSAIDS stopped	0	11 (40.7)	**<0.01**
Biologic initiated	18 (43.9%)	3 (11.1%)	**<0.01**
Biologic switched	1 (2.4)	11 (40.7)	**<0.01**
Biologic increased dosage	3 (7.3)	1 (3.7)	1.0

Data are n (%) or median (IQR). *p*-value < 0.05 are bold. IBD = Inflammatory bowel disease. CD = Crohn’s disease. UC = Ulcerative colitis. 5-ASA = 5-aminosalicylic acid. NSAID = Non-steroidal anti-inflammatory drugs. CRP = C-reactive protein. HBI = Harvey–Bradshaw index. SCCAI = Simple Clinical Colitis Activity Index. AS = Ankylosing spondylitis.

**Table 3:** Baseline characteristic comparison between patients who underwent biologic treatment modification after the second diagnosis to patients who did not.

Characteristics (n = 68)	Patients with Biologic Initiation/Switch/Increased Dosage (n = 37)	Patients without Biologic Treatment Modification (n = 31)	*p*-Value
Male gender	27 (73)	13 (42)	0.01
Age, years	39 (28–47)	43 (33–57)	0.48
Smoking, current or former	9 (24.3)	13 (42)	0.64
Age at IBD diagnosis, years	27 (22–36)	33 (22–50)	0.1
Interval between the two diagnoses, years	5 (2–11)	3 (1–11)	0.62
CD	28 (75.7)	25 (80.6)	0.77
CD extent, n (% of CD patients)			0.67
L1 (ileal)	16 (57.1)	17 (68.0)	
L2 (colonic)	4 (14.3)	2 (8.0)	
L3 (ileo-colonic)	8 (28.6)	6 (24.0)	
CD perianal disease, n (% of CD patients)	6 (21.4)	1(4.0)	0.1
CD phenotype, n (% of CD patients)			0.3
B1 (non-structuring non-penetrating)	22 (78.6)	20 (80.0)	
B2 (structuring)	2 (7.1)	4 (16.0)	
B3 (penetrating)	4 (14.3)	1 (4.0)	
UC extent, n (% of UC patients)			0.05
E1 (proctitis)	0	2 (33.3)	
E2 (left-sided)	4 (44.4)	1 (16.7)	
E3 (extensive)	5 (55.6)	3 (50.0)	
Clinical extraintestinal manifestation			
Psoriasis	3 (8.1)	0	0.245
Uveitis	4 (10.8)	3 (9.7)	1.0
Peripheral arthritis	11 (29.7)	5 (16.1)	0.23
CRP at time of second diagnosis	14.5 (5–42)	29.0 (10–70)	0.58
IBD activity at time of second diagnosis			0.46
Low	10 (27.0)	13 (41.9)	
Moderate	14 (37.8)	11 (35.5)	
High	12 (32.4)	7 (22.6)	
Very high	1 (2.7)	0	
AS activity at time of second diagnosis			0.92
Low	3 (8.1)	2 (6.5)	
Moderate	14 (37.8)	13 (41.9)	
High	20 (54.1)	16 (51.6)	
Previous biologic therapy	8 (11.8)	9 (13.2)	0.37
Ongoing treatment during second diagnosis			
Biologic regimen	16 (43.2)	10 (32.3)	0.45
Corticosteroids	4 (10.8)	3 (9.6)	0.68
5-ASA	6 (16.2)	8 (25.8)	0.33
Immunomodulators (azathioprine/mercaptopurine/MTX)	4 (10.8)	3 (9.6)	0.67
NSAIDS	5 (13.5)	7 (22.6)	0.26

Data are n (%) or median (IQR). *p*-value < 0.05 are bold. IBD = Inflammatory bowel disease. CD = Crohn’s disease. UC = Ulcerative colitis. 5-ASA = 5-aminosalicylic acid. NSAID = Non-steroidal anti-inflammatory drugs. MTX = Methotrexate. CRP = C-reactive protein. AS = Ankylosing spondylitis.

**Table 4:** Comparison of IBD and AS improvement rates 3 months after biologic modification, stratified by first diagnosis.

	IBD Improvement at 3-Month Follow-Up Visit			AS Improvement at 3-Month Follow-Up Visit			Either IBD or AS Improvement at 3-Month Follow-Up Visit		
	n/N (%) in patients with biologic treatment modification (initiation/switch/increased dosage)	n/N (%) in patients without biologic treatment modification	*p*-value	n/N (%) in patients with biologic treatment modification (initiation/switch/increased dosage)	n/N (%) in patients without biologic treatment modification	*p*-value	n/N (%) in patients with biologic treatment modification (initiation/switch/increased dosage)	n/N (%) in patients without biologic treatment modification	*p*-value
IBD first then AS (n = 41)	8/22 (36.4%)	0/19 (0.0%)	0.003	13/22 (59.1%)	6/19 (31.6%)	0.078	16/22 (72.7%)	6/19 (31.6%)	0.008
AS first then IBD (n = 27)	8/15 (53.3%)	3/12 (25.0%)	0.137	4/15 (26.7%)	2/12 (16.7%)	0.535	9/15 (60.0%)	4/12 (33.3%)	0.168
Overall (n = 68)	16/37 (43.2%)	3/31 (9.7%)	0.002	17/37 (45.9%)	8/31 (25.8%)	0.086	25/37 (67.6%)	10/31 (32.3%)	0.004

IBD = Inflammatory bowel disease. AS = Ankylosing spondylitis.

**Table 5:** Univariate and multivariate analysis of the possible predictors of improvement in IBD or AS at 3 months after the second diagnosis.

	Univariate			Multivariate		
Characteristic	Odds Ratio	95% Confidence Interval	*p*-Value	Odds Ratio	95% Confidence Interval	*p*-Value
Male gender	1.8	0.68–4.79	0.236			
Smoking	1.58	0.57–4.40	0.386			
Interval between the two diagnoses (cut-off = 4 years)	1.11	0.42–2.91	0.839			
CD vs. UC	1.81	0.57–5.82	0.317			
IBD vs. AS first diagnosis	1.25	0.47–3.29	0.657			
Treatment was changed at time of second diagnosis	1.07	0.28–4.10	0.92			
Biologic treatment modification (initiation/switch/increased dosage)	4.38	1.58–12.14	0.005	3.69	1.08–12.58	0.037
Current biologic treatment	3.14	1.02–9.65	0.046	1.27	0.32–5.130	0.736
Current corticosteroids treatment	2	0.72–5.53	0.182			
IBD severity at time of second diagnosis	3	0.99–9.13	0.053	2.64	0.80–8.650	0.108
AS severity at time of second diagnosis	1.12	0.43–2.89	0.819			

*p*-value < 0.05. CD = Crohn’s disease. UC = Ulcerative colitis. IBD = Inflammatory bowel disease. AS = Ankylosing spondylitis.

## Data Availability

Data is available upon special request however anonymity is maintained.
